# Toward the Drosophila connectome: structural analysis of the brain network

**DOI:** 10.1186/1471-2202-14-S1-P63

**Published:** 2013-07-08

**Authors:** Chi-Tin Shih, Olaf Sporns, Ann-Shyn Chiang

**Affiliations:** 1 Department of Physics, Tunghai University, 40704 Taichung, Taiwan; 2 Department of Psychological and Brain Sciences, Indiana University, Bloomington, IN 47405, USA; 3 Brain Research Center, National Tsing Hua University, 30013 Hsinchu, Taiwan

## 

The brain can be conceptualized as a complex network. It is believed that the topological structure of the brain network is closely related to the functions of the brain [1]. Therefore, understanding the network structure of the brain is a crucial task in neuroscience. In this report, we propose a first draft of the network architecture of the *Drosophila *connectome at the mesoscopic scale. The structural network of the *Drosophila *brain is constructed from a dataset of more than 20,000 single neurons in female brain assembled in the FlyCircuit database (http://www.flycircuit.tw)[2], the most comprehensive database of single-neuron images of the *Drosophila *brain to date. The nodes of the network represent mesoscopic brain regions called *Local Processing Units *(LPUs). The weight of the edges connecting each node pair corresponds to the number of neurons innervating the two LPUs reciprocally. The network shows hierarchical structure, pronounced small-world characteristics with high clustering and high global efficiency, and it is composed of six modules corresponding to known functional domains including the sensory modalities (including olfactory, mechano-auditory, and visual), together with the pre-motor and motor centers. Based on the modular structure of the network, we propose two models for the flow of information associated with intuitive and reasoning behaviors, respectively.
